# The effects of post-operative oxygen supply on blood oxygenation and acid-base status in rats anaesthetized with fentanyl/fluanisone and midazolam

**DOI:** 10.1371/journal.pone.0255829

**Published:** 2021-08-09

**Authors:** Leander Gaarde, Stefanie Kolstrup, Peter Bollen

**Affiliations:** 1 Department of Cardio-Renal Research, Institute of Molecular Medicine, University of Southern Denmark, Odense, Denmark; 2 Biomedical Laboratory, Institute of Molecular Medicine, University of Southern Denmark, Odense, Denmark; Royal College of Surgeons in Ireland, IRELAND

## Abstract

In anaesthetic practice the risk of hypoxia and arterial blood gas disturbances is evident, as most anaesthetic regimens depress the respiratory function. Hypoxia may be extended during recovery, and for this reason we wished to investigate if oxygen supply during a one hour post-operative period reduced the development of hypoxia and respiratory acidosis in rats anaesthetized with fentanyl/fluanisone and midazolam. Twelve Sprague Dawley rats underwent surgery and were divided in two groups, breathing either 100% oxygen or atmospheric air during a post-operative period. The peripheral blood oxygen saturation and arterial acid-base status were analyzed for differences between the two groups. We found that oxygen supply after surgery prevented hypoxia but did not result in a significant difference in the blood acid-base status. All rats developed respiratory acidosis, which could not be reversed by supplemental oxygen supply. We concluded that oxygen supply improved oxygen saturation and avoided hypoxia but did not have an influence on the acid-base status.

## Introduction

It is well known that oxygen supply during surgery in rodents improves post-operative recovery. In rats anaesthetized by ketamine and xylazine, oxygen supply during the procedure reduced mortality from 58% to 17% during bile duct ligation [1]. As a reason for mortality, drug induced hypoxia was suggested. Xylazine has a detrimental effect on oxygenation of the peripheral blood, as it induced pulmonary oedema with a rapid onset, cellular damage and pleural fluid, in combination with a transient hypertension, followed by hypotension, bradycardia and heart block [2, 3]. Other drug combinations may also cause hypoxia, such as combinations containing the opioids fentanyl or sufentanil. Opiates cause respiratory depression, which can be seen by a reduced respiratory rate and low blood oxygen saturation [4]. For this reason, pulsoximetry should always be performed during surgical procedures in rodents for monitoring vital signs during anaesthesia [5]. Moreover, acid-base status of anaesthetized animals can be monitored by analyses of arterial blood samples, which is valuable during long periods of anaesthesia and when normal physiological parameters are important for the outcome of the study [5]. In rats, arterial blood samples can be obtained by catheter placement in the tail artery [6, 7].

As reported in other studies [1, 4] and our own experiences in a rat model for abdominal aortic aneurysm, oxygen supply prevents hypoxia during the surgical procedure. We wished to investigate if prolonged oxygen supply during a post-operative period of one hour, further improved blood oxygenation and if this had an influence on the arterial acid-base status.

## Materials & methods

Twelve male Sprague Dawley rats (RjHan:SD, Janvier, France) with a body weight of 365 ± 14 g were anaesthetized by a subcutaneous injection of fentanyl (236 μg/kg), fluanisone (7.5 mg/kg) and midazolam (3.75 mg/kg) diluted with sterile water, according to the standard protocol of the facility. Prior to anaesthesia, the animals were housed in IVC cages (Allentown Rat 1800 cage) at an environmental temperature of 21 ± 1 ^o^C, relative humidity of 45–65% and 12h light/dark cycle. Food and water were available ad libitum, and animals were not fasted prior to surgery.

The anaesthetic depth was examined by an interdigit pinch with a hemostat at first click, to confirm absence of the paw withdrawal reflex and adequate surgical anaesthesia was obtained. A laparotomy was performed, and intestines were carefully removed from the abdomen and wrapped in gauze soaked in saline, to expose the abdominal aorta. After 10 minutes, the intestines were replaced and the abdomen was closed with a 4.0 Vicryl suture for muscle layer and 5.0 Vicryl suture for skin layer. All rats received 0.8 L/min 100% oxygen supplied through a nose mask during surgery. The entire procedure lasted approximately 20 minutes. During surgery, animals were maintained on a heating pad adjusted to 38°C (Physio Suite, Kent Scientific, USA). No additional analgesics were administered.

Immediately after surgery blood oxygen saturation and heart rate were measured with a pulse oximeter (Mousestat Junior, Kent Scientific, USA). Respiratory rate was measured by visual inspection and counting the number of breaths pr. minute, and temperature was measured by a rectal probe (Physio Suite, Kent Scientific, USA). An arterial blood sample (90 μL) was obtained by percutaneous catheterization of the tail artery with a 27G Neoflon over-the-needle catheter for blood gas analysis while the rats were still receiving oxygen. The blood samples were immediately analyzed (epoc® Blood Analysis System, Siemens, Germany). Parameters included were pH, pCO_2_, HCO_3_^-^ and pO_2_.

After surgery, the rats were randomly assigned in two groups and placed in a heated cabinet (26°C), in a Macrolon type III cage with Aspen bedding material. One group received 0.25 L/min oxygen via a nose mask and one group was left without oxygen supply, breathing atmospheric air (n = 6 for each treatment). During the post-operative period, 30 and 60 minutes after surgery, oxygen saturation, heart rate, respiratory rate and rectal temperature were measured and 60 minutes after surgery an arterial blood sample was taken for immediate analysis of the acid-base status. After this, the animals were euthanized with a 1.0 ml intraperitoneal injection of pentobarbital 400mg/ml, while still in anaesthesia.

The study was ethically approved by the Danish Animal Experiments Inspectorate (license nr. 2016−15−0201−00816). All surgery was performed under fentanyl/fluanisone and midazolam anaesthesia, and all efforts were made to minimize suffering.

Statistical analyses were performed with GraphPad PRISM 6.0 (GraphPad software, San Diego, USA). Data was examined for normal distribution and analysed with ANOVA. Multiple comparisons were performed using Tukey’s post hoc test. Baseline data for both groups measured immediately after surgery was analysed by a two-tailed unpaired parametric student’s t-test. A *p*-value <0.05 was considered statistically significant.

## Results

In all animals (n = 12) the oxygen saturation, heart rate, respiratory rate and body temperature were measured immediately after surgery, as well as after 30 and 60 minutes during the post-operative period. No differences between groups were found for baseline data measured immediately after surgery (*p*>0.05).

**Fig 1:**
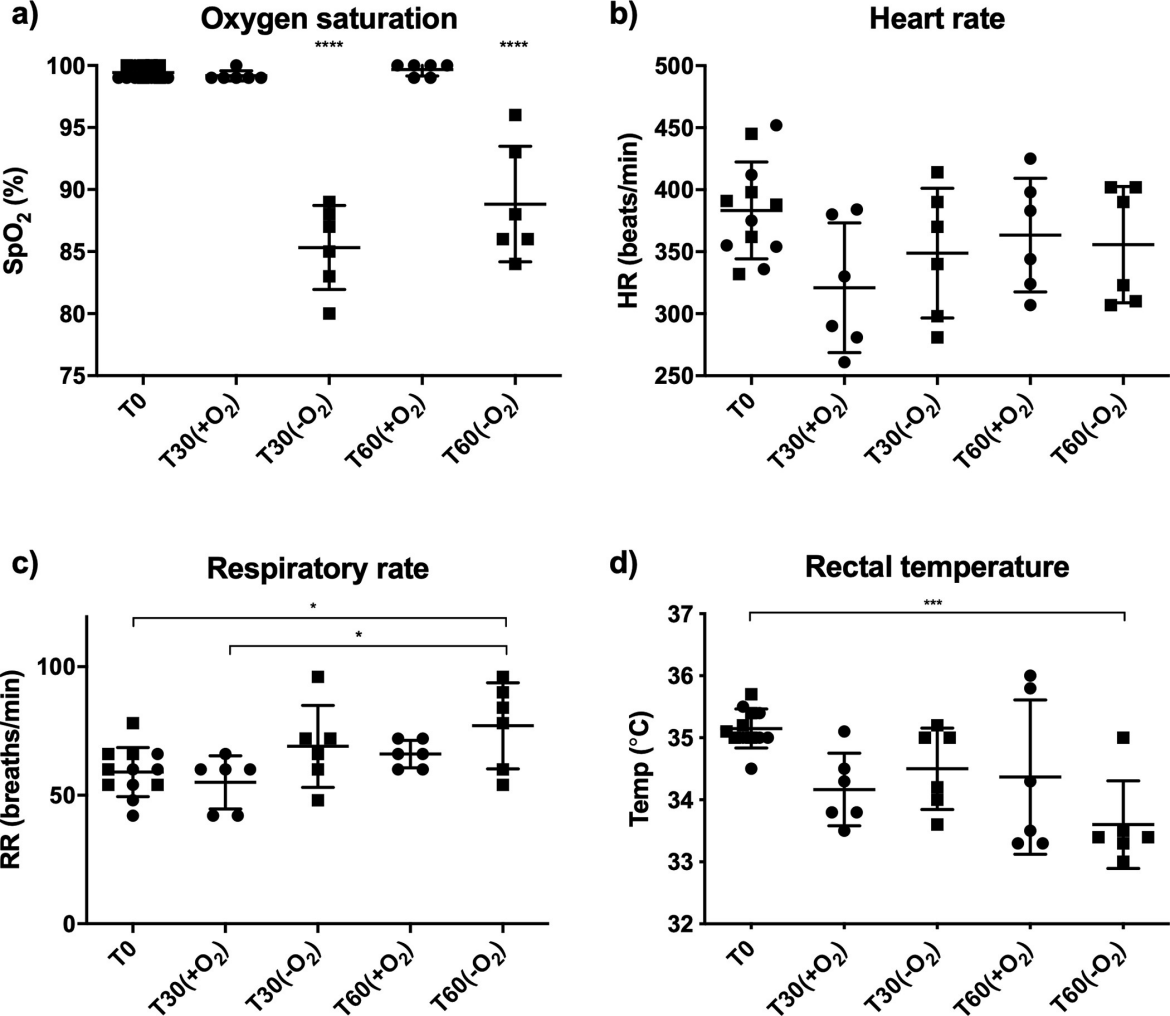
Vital signs with oxygen saturation (a), heart rate (b), respiratory rate (c) and rectal temperature (d) of rats after surgery under fentanyl/fluanisone and midazolam anaesthesia. T0 represents measurements immediately after surgery. Measurements 30 and 60 minutes after surgery are indicated by T30 or T60 respectively. Oxygen supply is indicated by +O_2_ (with oxygen supply) and -O_2_ (without oxygen supply). Symbol shape indicates +O_2_ group for circle and -O_2_ group for square. In (a) **** indicates *p*<0.0001 for T30(-O_2_) and T60(-O_2_) versus all other measure points. In (c) * indicates *p*<0.05 for T0 versus T60(-O_2_) and T30(+O_2_) versus T60(-O_2_), and in (d) *** indicates p<0.001 for T0 versus T60(-O_2_). HR: Heart rate, RR: Respiratory rate, Temp: Temperature. Values are means **±** standard deviations.

Fig 1 displays these results with indication of the level of significance. Significant differences were detected in oxygen saturation and respiratory rate; oxygen saturation was lower (*p*<0.001) 30 and 60 minutes after surgery without oxygen supply compared to all other measured values. The respiration rate was higher (*p*<0.05) 60 minutes after surgery without oxygen supply compared to values immediately after surgery and 30 minutes after surgery with oxygen supply.

**Fig 2:**
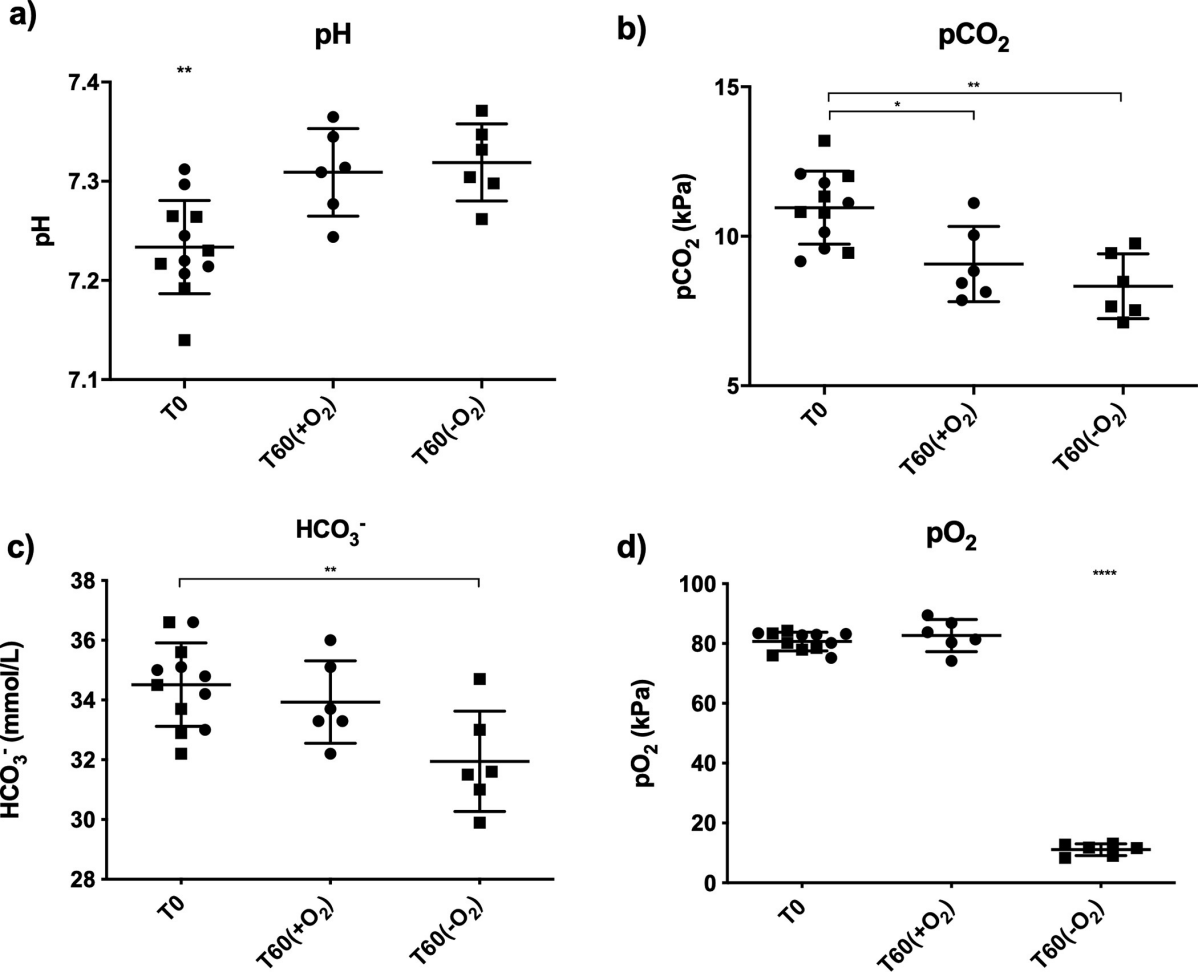
Blood acid-base status with pH (a), pCO_2_ (b), HCO_3_^-^ (c) and pO_2_ (d) of rats after surgery under fentanyl/fluanisone and midazolam anaesthesia. T0 represents measurements immediately after surgery. Measurements 60 minutes (T60) after surgery are indicated by +O_2_ (with oxygen supply) and -O_2_ (without oxygen supply). Symbol shape indicates +O_2_ group for circle and -O_2_ group for square. In (a) ** indicates *p*<0.01 for T0 versus T60(+O_2_ and -O_2_). In (b) * indicates *p*<0.05 for T0 versus T60(+O_2_) and ** *p*<0.01 for T0 versus T60(-O_2_). In (c) ** indicates *p*<0.01 for T0 versus T60(-O_2_) and in (d) **** indicates p<0.0001 for T60(-O_2_) versus T0 and T60(+O_2_). Values are means **±** standard deviations.

The results from the arterial blood samples analyzed immediately after surgery and 60 minutes after surgery are presented in Fig 2. No differences between groups were found for baseline data measured immediately after surgery (*p*>0.05). Significant differences were detected, as the pH value was lower immediately after surgery (*p*<0.01) compared to values after 60 minutes with and without oxygen supply, and pCO_2_ was higher immediately after surgery compared to 60 minutes after surgery with oxygen (*p*<0.05) and without oxygen supply (*p*<0.01). HCO_3_^-^ was higher immediately after surgery compared to after 60 minutes without oxygen supply (*p*<0.01). Finally, pO_2_ was lower in rats 60 minutes after surgery without oxygen supply (*p*<0.001) compared to all other groups.

## Discussion

In this study, we aimed to investigate if post-operative oxygen supply had an influence on blood oxygenation and arterial acid-base status in rats anaesthetized with fentanyl/fluanisone and midazolam to advance knowledge on physiological parameters during anaesthesia of laboratory rat surgery. We found that post-operative oxygen supply at 0.25 L/min significantly reduced hypoxia, but it did not prevent respiratory acidosis. Blood oxygen saturation of rats receiving oxygen after surgery was >99%, but in rats breathing atmospheric air the mean blood oxygen saturation was <90% 30 and 60 minutes after surgery, which was considered as hypoxic [8, 9].

It is well known that anaesthesia can reduce the respiratory rate, causing hypoxia [4], but little notice has been paid to the importance of post-operative oxygen supply, nor has the acid-base status been evaluated during this period. Picollo et al. described the cardiovascular effects of xylazine/ketamine after anaesthesia in rats and concluded that aversive cardiovascular and thermoregulatory effects persist during recovery [10]. With respect to respiratory parameters, our findings are in line with this finding. Hypoxia persisted in rats breathing atmospheric air 60 minutes post-operatively. Hypoxia after anaesthesia with opioid drug combinations is caused by a depressed respiratory function as shown by Hedenqvist et al., who used combinations of sufentanil/medetomidine and observed a 50% decrease in respiratory rate [4]. This effect is enhanced when anaesthesia is prolonged by supplemental injections [5, 11]. Hypoxia leads to an 8-hour delayed onset of pulmonary oedema [3], emphasizing the importance of avoiding hypoxia during and after anaesthetic protocols. In our study, significant low oxygen saturation 30 and 60 minutes after surgery in rats not receiving oxygen confirm this effect. Our findings contribute to existing knowledge, as hypoxia can be easily treated by increasing the inspired oxygen concentration. Our study shows that severe hypoxia occurs if supplemental oxygen is not provided during the post-operative period.

The partial CO_2_ tension in arterial blood was similar in rats receiving oxygen as in rats breathing atmospheric air, but both groups had values almost two times higher than reference values from awake rats [12, 13]. An increased partial CO_2_ tension leads to a decrease in arterial blood pH and the development of respiratory acidosis, as seen in our study. Therefore, our findings indicate that while hypoxia can be counteracted by supplemental oxygen supply, hypercapnia persists, causing a change in physiological parameters during the post-operative period. This finding contributes to existing literature, as consistent physiological parameters among groups are of great importance when planning experimental laboratory protocols, and researchers are encouraged to address whether acidosis affects experimental outcomes, as acidosis cannot be reversed by supplemental oxygen supply. When respiratory acidosis is present, assisted ventilation may be introduced to correct the acid-base imbalance [8].

The relation between blood gas tensions and acid-base status during various combinations of anaesthesia in rats is a well described mechanism and has been of focus for many years [14]. In our study, blood gas parameters showed respiratory acidosis with low pH values and accumulation of CO_2_. The partial O_2_ tension in rats not receiving oxygen was significantly lower 60 minutes after surgery and was in line with the low oxygen saturation of the blood. Although the partial CO_2_ tension was twice as high as the reference values immediately after surgery [5, 11], this was reduced significantly after 60 minutes in combination with an increased pH, slowly normalizing the respiratory acidosis.

Body temperature affects blood gas parameters due to the increase and decrease of gas solubility during hypo- and hyperthermia respectively [15]. In our study, a mild degree of hypothermia was observed in all rats, although more severely in rats not receiving oxygen 60 minutes after surgery. Hypothermia is known to cause an increase in pH due to a decrease in partial CO_2_ tension [16]. Moreover, a leftward switch in the oxyhemoglobin dissociation curve is caused by hypothermia due to an increase in hemoglobin-oxygen affinity [17]. Thus, the actual severity of observed hypoxia and respiratory acidosis in rats not receiving oxygen after surgery could be masked by the effects of hypothermia on blood acid-base status.

In conclusion, our results indicate that administration of oxygen during the post-operative period can prevent hypoxia but does not result in a significant difference in the blood acid-base status. Thus, the respiratory acidosis is unaffected by oxygen supply. However, we recommend supplementing with oxygen after anaesthetic procedures, to improve oxygen saturation and avoid hypoxia.

## Supporting information

S1 Table Vital signs of rats under fentanyl/fluanisone and midazolam anaesthesia with and without oxygen during a post-operative period of one hour. (PDF)

S2 Table Blood acid-base status of rats under fentanyl/fluanisone and midazolam anaesthesia with and without oxygen during a post-operative period of one hour. (PDF)

## References

[1] Mechelinck M, Kupp C, Krüger JC, Habigt MA, Helmedag MJ, Tolba RH, et al. Oxygen inhalation improves postoperative survival in ketamine-xylazine anaesthetised rats: An observational study. PLoS ONE. 2019;14(12):e0226430. doi: 10.1371/journal.pone.0226430 31834913 PMC6910690

[2] Amouzadeh HR, Sangiah S, Qualls CW, Cowell RL, Mauromoustakos A. Xylazine-Induced Pulmonary Edema in Rats. Toxicology and Applied Pharmacology. 1991;108:417–427. doi: 10.1016/0041-008x(91)90088-v 1902333

[3] Rassler B, Marx G, Reissig C, Rohling MA, Tannapfel A, Wenger RH, et al. Time course of hypoxia-induced lung injury in rats. Respir Physiol Neurobiol. 2007;159(1):45–54. doi: 10.1016/j.resp.2007.05.008 17597012

[4] Hedenqvist P, Roughan J, Flecknell PA. Sufentanil and medetomidine anaesthesia in the rat and its reversal with atipamezole and butorphanol. Laboratory Animals. 2000;34:244–251. doi: 10.1258/002367700780384762 11037117

[5] Tremoleda JL, Kerton A, Gsell W. Anaesthesia and physiological monitoring during in vivo imaging of laboratory rodents: considerations on experimental outcomes and animal welfare. EJNMMI Res. 2012;2(1):44. doi: 10.1186/2191-219X-2-44 22877315 PMC3467189

[6] Balla DZ, Schwarz S, Wiesner HM, Hennige AM, Pohmann R. Monitoring the stress-level of rats with different types of anesthesia: a tail-artery cannulation protocol. J Pharmacol Toxicol Methods. 2014;70(1):35–39. doi: 10.1016/j.vascn.2014.03.003 24632523

[7] Kumagai K, Horikawa M, Yamada K, Uchida BT, Farsad K. Transtail Artery Access in Rats: A New Technique for Repeatable Selective Angiography. J Vasc Interv Radiol. 2020;31(4):678–681. doi: 10.1016/j.jvir.2019.07.027 31706884

[8] Grimm KA, Lamont LA, Tranquilli WJ, Greene SA, Robertson SA. Veterinary Anesthesia and Analgesia, Fifth edition. Oxford: Wiley Blackwell, 2015

[9] Giroux MC, Santamaria R, Hélie P, Burns P, Beaudry F, Vachon P. Physiological, pharmacokinetic and liver metabolism comparisons between 3-, 6-, 12- and 18-month-old male Sprague Dawley rats under ketamine-xylazine anesthesia. Exp Anim. 2016;65(1):63–75. doi: 10.1538/expanim.15-0039 26489361 PMC4783652

[10] Picollo C, SerraII AJ, LevyI RF, AntonioI EL, dos Santos L, TucciI PJF. Hemodynamic and thermoregulatory effects of xylazine-ketamine mixture persist even after the anesthetic stage in rats. Arq Bras Med Vet Zootec. 2012;64(4):860–864. doi: 10.1590/S0102-09352012000400011

[11] Flecknell PA M Mitchell. Midazolam and fentanyl-fluanisone: assessment of anaesthetic effects in laboratory rodents and rabbits. Lab Anim. 1984;18(2):143–146. doi: 10.1258/002367784780891406 6748593

[12] Girard P, Brun-Pascaud M, Pocidalo JJ. Acid-base status of awake rats after cannulation of aorta and vena cava. Kidney Int. 1983; 24 (6): 795–799. doi: 10.1038/ki.1983.230 . 6674673

[13] Steiner AR, Rousseau-Blass F, Schroeter A, Hartnack S, Bettschart-Wolfensberger R. Systematic Review: Anaesthetic Protocols and Management as Confounders in Rodent Blood Oxygen Level Dependent Functional Magnetic Resonance Imaging (BOLD fMRI)–Part A: Effects of Changes in Physiological Parameters. Neurosci. 2020;14:577119. doi: 10.3389/fnins.2020.577119 33192261 PMC7646331

[14] Svendsen P, Carter AM. Influence of injectable anaesthetic combinations on blood gas tensions and acid-base status in laboratory rats. Acta Pharmacol Toxicol (Copenh). 1985;57(1):1–7. doi: 10.1111/j.1600-0773.1985.tb00001.x 3931415

[15] Bacher A. Effects of body temperature on blood gases. Intensive Care Med. 2005;31(1):24–27. doi: 10.1007/s00134-004-2369-3 15221130

[16] Alston TA. Blood gases and pH during hypothermia: the "-stats". Int Anesthesiol Clin. 2004;42(4):73–80. doi: 10.1097/00004311-200404240-00008 15577701

[17] Bisson J, Younker J. Correcting arterial blood gases for temperature: (when) is it clinically significant? Nurs Crit Care. 2006;11(5):232–238. doi: 10.1111/j.1478-5153.2006.00177.x 16983854

