# Supplementary material for: The effects of post-operative oxygen supply on blood oxygenation and acid-base status in rats anaesthetized with fentanyl/fluanisone and midazolam
Source: PLoS One. 2021 Aug 9;16(8):e0255829. doi: 10.1371/journal.pone.0255829 (PMC8351956; doi:10.1371/journal.pone.0255829)
Supplement: S1 Table — (PDF) [file pone.0255829.s001.pdf]

S1 Table. Vital signs of rats under fentanyl/fluanisone and midazolam anaesthesia with and without oxygen during a post-operative period of one hour.

	**T0**	**T30**		**T60**	
**Oxygen supply**	$+O_2$	$+O_2$	$-O_2$	$+O_2$	$-O_2$
**SpO_2 (%)**	99.4 ± 0.5%	99.2 ± 0.4	85.3 ± 3.4	99.7 ± 0.5	88.8 ± 4.6
**HR (BPM)**	383 ± 39	321 ± 52	349 ± 52	364 ± 46	356 ± 47
**RR (RPM)**	59 ± 10	55 ± 10	69 ± 16	66 ± 5	77 ± 17
**T (°C)**	35.2 ± 0.3	34.2 ± 0.6	34.5 ± 0.7	34.4 ± 1.25	33.6 ± 0.7

Means ± standard deviations. T0 indicates measurements immediately after surgery (n=12), T30: 30 minutes after surgery and T60: 60 minutes after surgery. $+O_2$: with oxygen supply (n=6). $-O_2$: without oxygen supply (n=6). HR: Heart rate, RR: Respiratory rate, T: Temperature, BPM: Beats per minute, RPM: Respirations per minute.
