# Supplementary material for: The effects of post-operative oxygen supply on blood oxygenation and acid-base status in rats anaesthetized with fentanyl/fluanisone and midazolam
Source: PLoS One. 2021 Aug 9;16(8):e0255829. doi: 10.1371/journal.pone.0255829 (PMC8351956; doi:10.1371/journal.pone.0255829)
Supplement: S2 Table — (PDF) [file pone.0255829.s002.pdf]

**S2 Table. Blood acid-base status of rats under fentanyl/fluanisone and midazolam anaesthesia with and without oxygen during a post-operative period of one hour.**

| | **T0** | **T60** | |
|---|---|---|---|
| **Oxygen supply** | $+O_2$ | $+O_2$ | $-O_2$ |
| **pH** | 7.23 ± 0.04 | 7.31 ± 0.04 | 7.32 ± 0.04 |
| **pCO2 (kPa)** | 10.96 ± 1.22 | 9.07 ± 1.26 | 8.33 ± 1.08 |
| **$HCO_3^-$ (mmol/L)** | 34.52 ± 1.4 | 33.93 ± 1.38 | 31.95 ± 1.68 |
| **$pO_2$ (kPa)** | 80.69 ± 3.14 | 82.69 ± 5.37 | 11.11 ± 1.96 |

Means ± standard deviations. T0 indicates measurements immediately after surgery (n=12), T30: 30 minutes after surgery and T60: 60 minutes after surgery. $+O_2$: with oxygen supply (n=6). $-O_2$: without oxygen supply (n=6).
